# Reply to Kaestner et al.: Activation of PIEZO1 is not significant for the passage of red blood cells through biomimetic splenic slits

**DOI:** 10.1073/pnas.2411469121

**Published:** 2025-01-02

**Authors:** Alexis Moreau, François Yaya, Huijie Lu, Anagha Surendranath, Anne Charrier, Benoit Dehapiot, Emmanuèle Helfer, Annie Viallat, Zhangli Peng

**Affiliations:** ^a^CNRS, Centre Interdisciplinaire de Nanoscience de Marseille, Turing Centre for Living Systems, Aix Marseille Université, Marseille 13009, France; ^b^Richard and Loan Hill Department of Biomedical Engineering, University of Illinois, Chicago, IL 60612; ^c^CNRS, Institut de Biologie du Développement de Marseille, Turing Centre for Living Systems, Aix Marseille Université, Marseille 13009, France

We thank the authors for acknowledging the seminal nature of our work (1).

We have not conclusively addressed the question of Piezo1 activation during splenic filtration in vivo. Our aim was to highlight the key mechanisms governing red blood cell (RBC) retention/passage in slits. In the discussion, we were cautious in only suggesting that no PIEZO1 effect is involved in RBC passage.

Kaestner et al. wrote that “Accumulating evidence shows salt solution…contain Ca^2+^” (2). We carefully examined the articles cited supporting this statement.

In ref. 3, the authors use a Gardos channel activator. They observe hyperpolarization and deduce contamination by 4 μmol Ca^2+^. However, they give neither the cause (residual Ca^2+^ from blood plasma?) nor the dosage. Moreover, at low Gardos activator concentrations, no hyperpolarization is observed at 4 μmol Ca^2+^. Finally, hyperpolarization is a slow process (time scale: 500 s).

In ref. 4, the authors use a PIEZO1 activator. They observe hyperpolarization and cite ref. 3 to explain it but provide neither cause nor assay showing contamination. However, they estimate a plausible residual 8 μmol-Ca^2+^ originating from the plasma of the blood sample.

Our experimental conditions differ radically from those described in these articles:

1) We did not use Piezo1 or Gardos activator that promote activation at subphysiological values.

2) We took special precautions to avoid Ca^2+^ contamination. First, we used a Ca^2+^-free commercial buffer prepared from distilled water (See supplier’s data sheet available on the Internet). Second, we diluted the initial 30-μL blood drop in 1 mL (50-fold dilution) prior to centrifugation. The pellet was diluted in 500 μL and centrifuged twice, i.e., two successive 25-fold dilutions before a final 100-fold dilution, so that the residual Ca^2+^ concentration is <1 nmol, far too low to activate Gardos.

3) The slit passage time is a fast process <100 ms.

**Fig. 1.**
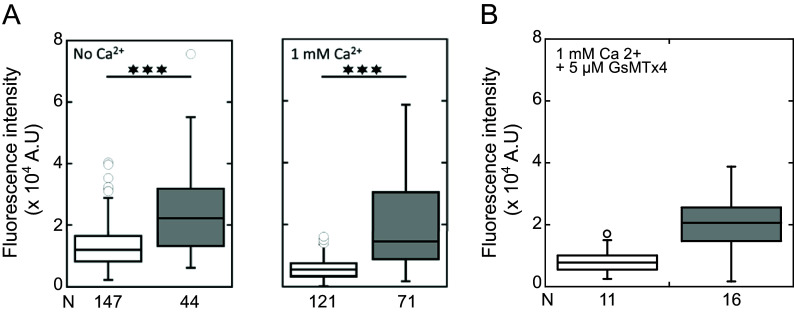
Intracellular calcium fluorescence intensity upstream (in white) and downstream of the slits (in gray) (*A*) in the absence or presence of 1 mM calcium in the buffer and (*B*) in the presence of 1 mM calcium in the buffer and of 5 µM GsMTX4, under ΔP = 500 Pa. Median values are displayed with 25% and 75% percentiles, and min/max values as whiskers. N: number of analyzed cells. Slit dimension: 0.80 × 2.77 × 4.70 µm^3^. Portions of this figure have been reused from Moreau et al. (1).

Finally, we performed a limited experiment using the PIEZO1 inhibitor (GsMTX4) and Fluo4, similarly to our paper’s figure 4 (reported here as Fig. 1*A*).

Although the statistics are limited, the trend is similar: i) an increase in intracellular Ca^2+^ signal after slit exit; ii) no difference in Ca^2+^ signal after exit, regardless of no Ca^2+^, or Ca^2+^ and PIEZO1 inhibitor in solution (Fig. 1*B*). Also, RBC transit times and velocities through the slits are unaffected by Ca^2+^ and/or PIEZO1 and Gardos inhibitors (Paper’s figures S9 and 5B). The similar transit times observed irrespectively of the presence of Ca^2+^ or PIEZO1 and Gardos inhibitors show that PIEZO1–Gardos channels do not induce a significant cell volume reduction, in agreement with a previous study (5). Thus, no significant change is expected in hemoglobin concentration and, consequently, in the Fluo4 signal.

Finally, our simulations show that the typical maximum membrane tension is about 421.23 pN/µm (figure 3F), below the critical tension (6) to activate PIEZO1 [1,440 pN/µm (7), 2,468 pN/µm (8), and 7,820 pN/µm (9)].

To conclude, we think that our experiments and conclusions are correct.

An experiment designed jointly by Kaestner et al. and ourselves would be beneficial in reaching a scientific consensus.
